# Bilateral emergency export reserve mechanism under climate change

**DOI:** 10.1186/s40066-021-00341-6

**Published:** 2022-04-09

**Authors:** Kuang-Sheng Huang

**Affiliations:** Graduate Institute of Industrial Economics, National Central University, Room 808, 8th Floor, Zhi-Xi Building, No. 300, Zhongda Rd., Zhongli District, Taoyuan City, 320317 Taiwan (R.O.C.)

**Keywords:** Climate change, Export restrictions, Bilateral emergency export reserves, Transnational agribusiness, Corporate social responsibility

## Abstract

Consider that multilateral food aid and regional food security mechanisms may incompletely adapt to the challenges of climate change in future practice. This study proposes a framework of bilateral emergency export reserve mechanism to encourage both participating countries to jointly appoint a transnational agribusiness to manage emergency export reserves as a means of fulfilling its corporate social responsibility (CSR). This mechanism has the features of simplified negotiation, more transparent operating procedures, along with reciprocity in bilateral cooperation, which would provide grain importing countries with a higher degree of safeguarding food security.

## Introduction

After the 2007–2008 food crisis, successive droughts in Russia and the United States have reduced grain production, causing the surge in global food prices during the period 2010–2012 [1, 2], and then gradually declining to 2015. Since then, global food prices have basically remained within a narrow range during the period 2015–2019, mainly due to good harvests in major grain exporting countries for several consecutive years. In particular, the rapid increase in Russian wheat production has made Russia the world’s largest wheat exporter since mid-2017. Further, India displaced Thailand in rice exports in 2012 and has taken a moderate leading position in subsequent years. Altogether, these have boosted the accumulation of staple grain stocks [3, 4], coupled with the United States' increase in shale oil and gas production to suppress a rise in international oil prices, which ultimately lowers operating costs for farmers. 

Global food prices have stabilized over a period of 5 years. This has led many consumers to experience the benefits of affordable food prices and have gradually weakened people’s awareness of the potential of recurring food crises. However, growing public concerns on food shortages spurred panic buying in the first half of 2020 due to pandemic disruptions in food supply chains, such as the suspension of production of food processing factories due to cluster infections and logistics disruptions caused by restrictions or lockdowns [5]. Fortunately, Food and Agriculture Organization of the United Nations (FAO) [6] predicted that world cereal production will reach record-highs in 2020 as the result of benefits from good weather conditions and higher yields of part of grain exporting countries, and the world cereals stocks-to-use ratio in 2020–2021 may hit a 20-year high before mid-2020. This may temporarily ease some public concerns about food insecurity. Nevertheless, Konandreas [7] mentioned that six episodes of soaring food prices have occurred since 1970, each lasting for about 2 years, totaling roughly one-third of the past 40 years. If ongoing global warming cannot be eventually controlled within 2 °C at the end of the twenty-first century, this would raise the risk of catastrophic climate change [8]. This, in turn, may cause negative impacts of climate change on agriculture, which may increase the frequency of food shortages due to production shortfalls of staple crops and would likely increase the risk of contemporary food crises in the future.

Over the past few decades, global food prices in nominal term have clearly trended upward since the 1970s, inflation-adjusted term began to reverse the downward trend in 2001 to increase steadily in later years. Prices soared during the period 2008–2011, and then maintained a somewhat stable level during the period 2015–2019 [9–11]. However, given the other factors discussed in this paper, it seems that an era of higher food prices is likely to be ahead. In the absence of a slowing rate of climate change, coupled with factors such as limited resources for agricultural production, population growth, and dietary shifts in developing countries resulting in an increase in per capita food demand [12]—we may expect growth in demand to frequently outstrip supply, which should eventually push up real food prices sustaining upward. This may have a significant impact on the fragile food security system of low-income food-deficit countries (LIFDCs), increases food import bills in net food-importing developing countries (NFIDCs), and may even have a perceptible effect on household spending in developed countries.

Grain exports from Russia and India have grown markedly in the last decade, increasing both countries’ influence on global food security. In recent history, Russia has sometimes experienced crop shortfalls due to abnormal drought, causing the country to purchase grains abroad in the early 1970s. Nearly four decades later, Russia has imposed a wheat export ban due to poor wheat harvests in 2010, which pushed up international wheat prices and had a profound impact on the economy and politics of North African countries [1, 13]. Further, Indian government-imposed rice export restrictions in 2007–2008 triggered a rice crisis [14]. Climate change may very likely increase the frequency of large-scale crop failures in major grain exporting countries, which may increase climate-induced food shortages and the possibility of some food-exporting countries imposing export restrictions. This will drive up global prices and long-term inflation expectations, increase monetary policy uncertainty of world’s major central banks, and further affect financial stability and global economic growth. In addition, the increased frequency of export restrictions will weaken the confidence of food-importing countries to rely on global grain trade (food self-reliance) as an improvement in food security.

Governments in some food-importing countries took countermeasures, though with limited effects, to reduce the impact of export restrictions imposed by major food-exporting countries, triggering public resentment and political unrest [15], forcing these countries to seek international food aid^1^ as a last resort in response to the emergency needs. However, if climate change exacerbates the food crisis in the future, that may increase the chance that the number of countries requesting assistance from WFP, and the amount of emergency food assistance required by WFP will exceed its ordinary assistance capacity for LIFDCs and NFIDCs. In addition, policymakers in major food donor countries have been cutting the foreign aid budget due to fiscal restraints, further limiting WFP funding from in-kind or cash contributions of major donators and affecting its operational capability.

The purpose of the regional food reserve mechanism is to improve regional food security by earmarked or stockpiled emergency food reserves of the organization’s member countries. However, earmarked emergency food reserves of the organization’s member countries may be insufficient to cope with large-scale disasters, and political struggles among major donor countries may undermine assistance capability. Further, this may be accompanied by the economic power and political influence exerted by larger participating countries on regional countries. There are additional concerns that some of the organization’s member countries have a free-riding mentality, and that food-exporting countries will be concerned about the impact of regional food reserves on free trade in grains, thus reducing the effectiveness of the mechanism during food crises.

Progress in strengthening multilateral agreements on export restrictions has been stagnant since the 2007–2008 food crisis. In addition, major food-importing countries encountered challenges in improving national food security. This study proposes to utilize a bilateral emergency export reserve mechanism with transnational agribusiness as a carrier to address the possible outcomes of climate change. This mechanism would have the properties of bilateral cooperation and mutual benefit with the primary goal of enhancing a safety network through international trade relations between grain importing and exporting countries. The following sections discuss two substantial determinants of food security including climate change and export restrictions, practices and challenges of developing Asian countries in improving their own national food security after the 2007–2008 food crisis, the roles of transnational agribusiness in food security, and the later section proposes a bilateral emergency export reserve mechanism. Conclusions and future research directions are in the last section.

## Two substantial determinants of affecting global food security

Global food security is affected by natural and man-made disasters. Climate change predictions suggest to increase the number and severity of natural disasters with likelihood of food crises in the future, and trade restrictions further exacerbate the severity of each food crisis by limiting supply as described below.

### Climate change influences global food security in the long term

The major impact of climate change on food supply is likely to slow down the growth rate and increase the instability of grain production, while higher average temperatures will shorten the growing season and reduce crop yields. An accelerated pace of climate change would have a greater negative impact on crop yields because of the non-linear relationship between yields and biophysical stresses. Once the temperature surpasses a critical threshold, crops will suffer severe damage and lead to a rapid decline in output [18].

With increasing scarcity of water resources, the shrinkage of agricultural land caused by industrialization, urbanization, environmental pollution and land degradation, coupled with the occurrence and severity of plant diseases and insect pests, as well as more severe natural disasters caused by weather extremes, such as forest fires, sand and dust storms, erratic rainfall in some areas, as well as hurricanes and floods, there is a possibility that sea level rise will accelerate [19]. Meanwhile, this could be accompanied by biodiversity losses, anthropogenic air pollution and haze damage, these may lead to reduced crop yields [20, 21]. As a result, the potential exists that the growth of food supply may be unable to meet demand, which would further push up global food prices in the long term.

In addition to weakening the food security system in the Horn of Africa and the Middle East that are recurring famine and malnutrition, the food supply in India and Southeast Asia will be the most vulnerable regions affected by climate change [8, 22]. The severely impact of climate change on crop yields is not limited in low-income and food-insecure countries, even the growing regions of major food-exporting countries, such as North America, South America, the Black Sea region and Australia may face a slowdown in agricultural productivity growth [22, 23].

Although the cultivation areas in high-latitude countries may be benefited by climate change, local planting conditions will be a factor. For instance, the poor soil condition in northern Russia makes it impossible to simply migrate wheat production to the northern part of the country to offset crop yield reductions in lower latitudes. There is also a possibility of breeding new varieties through genetic modification, which would allow those crops to adapt to weather extremes, but they will still face biophysical limits [22]. Unless those issues are addressed, we should expect to see a tight supply–demand balance of staple foods.

If we would continually see more extreme climate events and disasters caused by accelerating climate change, such as droughts, floods, heat waves, and ENSO extremes, these would gradually increase the systemic risk of production shocks. In the second half of the twentieth century, a once-in-a-century production shock event occurred. It is estimated that such shocks may occur as a once-in-30-year event by 2050, a risk increase of more than threefold. The worst-case scenario is that two successive critical harvests may be lost in major grain production areas, which could lead to an unprecedented systemic crisis [22, 24, 25]. Therefore, the risk of climate change threatens the living environment and food security as well as potentially resulting in climate refugees that the international community will face in the future [26].

In recent history, there have been several grain price spikes with some regularity, approximately every 30 years [27]. A long-term upward trend of grain prices will likely trigger the rise in short-term price peaks and medium-term price volatility, with the degree of correlation among these factors becoming higher [28]. Despite the fact that a long-term rise in grain prices would be expected to encourage farmers to increase planting, the increase in price volatility may offset the desirability of increased grain output. If the acceleration of climate change leads to an increase in the scale and duration of natural disasters, that could increase the possibility of steeper and even higher price spikes before gradually returning to pre-crisis levels at a slower rate than before. This would mean that the grain production capacity would not likely be restored to the pre-disaster scale immediately, thus potentially increasing panic buying for staple foods and impeding normal global grain trade works.

### Export restrictions exacerbate the severity of each food crisis

Policymakers in some grain exporting countries imposed export restrictions^2^ due to concerns of national food insecurity so as to meet domestic demand and stabilize food prices, ease inflationary pressure, and even increase fiscal revenue [14, 29]. However, export restrictions are beggar-thy-neighbor measures that will trigger soaring international food prices and exacerbate price fluctuations in international food markets. Perversely, this may cause neighboring countries and other grain exporting countries to impose even greater export restrictive measures afterwards, which in turn would deepen food insecurity during the food crisis.

When grain importing countries are confronted with rising food prices, this can cause strong public resentment and tremendous pressure from social unrest [15, 30]. Policymakers in these countries may have to take countermeasures including reducing food import tariffs, imposing food price controls and food subsidies, releasing grain stocks, restricting exports of locally-grown crops, and purchasing grains through government-to-government (G2G) deals. However, the increase in public expectations of food shortages and panicked hoarding may deepen anxieties about food insecurity and become a self-fulfilling prophesy. This could, in turn, force governments to take more stringent measures such as imposing purchase limits on certain foods. This would likely further trigger rampant black-market transactions and smuggling.

If grain exporting countries impose export restrictive measures and grain importing countries lower tariffs to increase imports, this may further drive up international food prices, causing the final results in trade volumes to be less significant [31]. FAO reported that during the 2007–2008 food crisis, roughly one-quarter of the surveyed countries imposed some forms of export restrictions, and about half cut food import taxes. However, the LIFDCs have limited scope to further reduce import tariffs on imported foods due to lower tariffs. Tariff reductions only partially counteract a rise in international food prices, but significantly reduce customs revenue for these countries [32, 33].

Taking the 2007–2008 rice crisis as an example, the thinness in the international rice market^3^ and a high concentration of exporters make it more vulnerable to the tight supply-demand balance [35]. This was a man-made crisis primarily caused by government policies, such as export restrictions imposed by some rice-exporting countries, and precautionary purchases by major rice-importing countries, causing international rice prices to rise more than other staple grains [36, 37]. Moreover, Headey [38] estimated that during the 2007–2008 rice crisis, the rice export restrictions imposed by India, Vietnam, Egypt and China accounted for 61% of the surge in international rice prices.

Notwithstanding that Indian policies may have started the 2007–2008 rice crisis, the crisis stemmed in part from poor wheat harvests in Australia in 2005–2006 [39], which led to a decline in world wheat stocks. The Indian government increased wheat imports and restricted rice exports afterwards to maintain its food security. Vietnam joined the ranks of rice export restrictors after India, and then the Philippines took precautions to purchase rice from Vietnam through G2G deals. In addition, some developing Asian countries such as China and Indonesia imposed country-specific countermeasures to reinforce national food security, resulting in domestic rice prices being more stable than international rice prices [40, 41]. These were the main factors driving a further rise in international rice prices. Slayton and Timmer [42] suggested that China, Japan and Thailand should be persuaded to release excess rice stocks to the international rice market to relieve the 2007–2008 rice crisis. Subsequently, the Government of Japan agreed that part of its surplus World Trade Organization (WTO) rice reserves would be exported to the Philippines,^4^ this exerted some restraining effect on international rice prices [27, 43].

After the 2007–2008 food crisis, some experts and scholars in agricultural trade policies proposed to strengthen WTO disciplines on export restrictions and to reduce the asymmetry in WTO regulations, mainly aimed at Article XI “General Elimination of Quantitative Restrictions” of GATT 1994, and Article 12 “Disciplines on Export Prohibitions and Restrictions” of the Agreement on Agriculture (AoA) [14, 29].

The regulatory deficiency or under-regulation of WTO provisions on export restrictions are as follows [14, 29]. First, if the export tax is unbound, a country imposes a sufficiently high tax that is equivalent to export prohibition and is compatible with WTO provisions. Secondly, the words of “temporarily”, “prevent”, “relieve” or “critical shortage” in paragraph 2(a) of Article XI of GATT 1994 are ambiguities that remain in the form of broad-ranging and legal interpretations. Third, the WTO has no clear corresponding penalty clauses for those countries to ignore the obligations of Article 12 of the AoA. Finally, the WTO does not clearly define “net food-exporting developing countries”, therefore, the WTO developing-country members have not absolutely complied with Article 12 of the AoA.

In order to remedy regulatory deficiency, Sharma [14] proposed two alternative schemes including a variable export tax scheme and a tax-rate quota. The variable export tax scheme is imposed by an automatic adjustment mechanism that precludes grain exporting countries arbitrarily raising export tax on their own. Despite its advantage of being simple and transparent, the variable export tax scheme is incompatible with WTO rules and would have required a rule change. This has little chance of acceptance by food importers especially during the period of soaring grain prices.

On the other hand, the tax-rate quota imposed by grain exporting countries in the same way as the tariff-rate quota (TRQ) will face how to set an appropriate quota, as well as the in-quota rate and the bound rate. Furthermore, grain exporting countries only need to activate tax-rate quota during the period of insufficient grain supply. However, as the urgency of exporting surplus grains in bumper harvest seasons, it becomes difficult to impose tax-rate quota regularly.

## Actions and challenges of developing Asian countries in reinforcing national food security

Asia is the most populous continent and Southeast Asia is one of the world’s most vulnerable regions to climate change. After the 2007–2008 food crisis, more developing Asian countries reinforced their national food security by boosting domestic production, adjusting national food self-sufficiency goals, and increasing the size of public grain reserves. They also sought to improve food self-reliance of some staple grains by promoting grain trade, as well as advocating and participating in regional cooperation for food security. But problems remain as follows.

The grain importing countries attempted to diversify their supplier base after the 2007–2008 food crisis, but that increased the cost of imported grains [29]. Additionally, despite the fact that regional trade agreements (RTAs) can generally be regarded as a form of WTO-Plus commitments, some countries within the region have frequently imposed export restrictions, making RTA unlikely to achieve stricter provisions on the export restrictions of WTO’s [29, 44, 45]. Further, the Uruguay AoA provisions regarding export restrictions are still vague and the Doha round has not corrected those. Trade protectionism is generally increasing worldwide. Therefore, it is very likely that export restrictions will be imposed by major grain exporting countries in any future food crisis to protect national food security, and the impact on global grain trade may gradually spread from rice to other staple grains.

Some Asian developing countries including China, Indonesia, and the Philippines intended to enhance national food security through adjusting food self-sufficiency goals^5^ after the 2007–2008 food crisis. In general, this is done through implementing guaranteed purchase prices and/or export subsidies, and reinforcing agricultural investment, such as development and maintenance of irrigation systems, promotion and exchange of cereal planting technology, adopting plant breeding technology for high-yield capacity, sustainable water resources management, improved farmland conservation and re-cultivation, even investing in agriculture overseas by acquiring or renting farmland to produce crops for their own domestic markets [46, 47, 49, 50]. However, there can be negative influences of such policies: (1) overemphasizing the importance of achieving food self-sufficiency goals may distort the efficiency of resource allocation, making the agricultural land development towards lower marginal production of soil resources, such as over-conversion of forest to agricultural land or excessive use of fertilizers and pesticides. There can also be a comparative disadvantage of such locally-produced foods which increases production costs and pushes up selling prices. This not only harms the domestic consumers’ interests, may as well deviate from the sustainability of agricultural development and eco-environmental protection in the long term [14, 51]; (2) guaranteed purchase prices aim to stabilize the livelihoods of local farmers and encourage crop cultivation. However, the continued reliance on trade-distorting domestic support, including the measures which support prices, could increase the fiscal burden of government, reduce the risk of farmers’ awareness of the need to improve/innovate, and increase the overproduction after harvests in these countries. In addition, in times of high international food prices, domestic farmers may be unable to make more profits abroad because governments impose these guaranteed purchase prices in conjunction with export restrictions. That could reduce their willingness to make additional agricultural investment both now and in the future [51]; and (3) foreign direct investment in agriculture through mergers and acquisitions of food manufacturers and/or traders in the food supply chain of investee countries may be disallowed by local governments due to perceptions of national security risk.

When the world wheat stocks-to-use ratio was at a relatively low level since 1970, that became the root cause of pushing up global wheat prices [11]. In 2001, the Committee on World Food Security recommended a threshold of 17–18% for staple grains [52]. In 2008, the world stocks-to-use ratios for wheat, maize and vegetable oils were at or near record lows since 1970, which triggered a global food crisis [53]. The reasons for a decline in world grain stocks in the past few decades have been a gradual reduction in trade barriers (which usually lowers costs) and relatively high storage costs (relative to the value of grain stocks), these caused many governments and private sectors around the world to reduce the size of their food reserves [10, 17, 54]. The 2007–2008 food crisis changed that mentality and induced many countries to aggressively accumulate public grain reserves during the period 2008–2010, despite increasing storage costs for those countries [55].

Managing public grain reserves (buffer stocks) is not an easy task, the difficulty lies in storage costs and how to construct transparent and effective rules to determine the best time to buy and sell stockpiles, regardless of whether a price band or a price floor is used for the operational strategy. For instance, the rules for properly determining upper and lower limits of the price band and trigger conditions have to be dynamically adjusted based on medium- to long-term trends and fluctuations in historical grain prices. Crucially, without sufficient funds, public grain reserves will eventually fail in theory and in practice [27, 29, 56].

The aim of the regional food reserve mechanism is to enhance food security in the organization’s member countries. However, insufficient total commitments by the organization’s member countries may limit the capability of the regional grain reserve scheme to respond to the emergency needs of large-scale natural disasters, partly because some member countries of the “free rider” mentality are perceived to be unwilling to contribute their “fair share”. Further, other grain exporters can be expected to resist such schemes due to conflicts of interest.

Taking ASEAN Plus Three Emergency Rice Reserve (APTERR) as an example, authorized representatives of the Association of Southeast Asian Nations (ASEAN) member countries, China, Japan, and South Korea have signed the APTERR agreement in October 2011. Although APTERR’s three programmes^6^ adopt earmarked or stockpiled emergency rice reserves, most of APTERR member countries received emergency assistance through Tier 3 until the end of 2019. Trethewie [58] pointed out that Tier 1 is the multilateral decision-making process for providing emergency triggering and releasing stockpiles in earmarked rice reserves as a source. These were supposed to be released based on the matching procedure between supplying and recipient countries by APTERR secretariat. Because of a lack of transparency in the procedure, potential recipient countries believed the benefits of seeking assistance through APTERR were not better than purchasing rice directly from the open market, thus reducing their willingness to use this programme for emergency needs.

In addition, the earmarked rice reserves of ASEAN member countries were much lower than those of the Plus Three countries (China, Japan, and South Korea). Some ASEAN member countries made commitments merely for diplomatic purposes and only provided the bare minimum required. This caused total earmarked rice reserves of APTERR to be insufficient for large-scale nature disasters. Thus expanding the amount of earmarked rice reserves will be a necessary measure to enhance the capability of APTERR, especially for major rice exporters, while those with limited rice production capacity will need to increase cash donations to APTERR [58, 59].

It is worth noting that climate change has gradually aggravated the severity of natural disasters, which may cause regional food shortages even more synchronized, and reduce the risk-sharing capability of countries in the region. Those countries may eventually require seeking assistance from outside the region. In addition, because APTERR is a regional rice reserve scheme, Southeast Asian countries are the major rice-exporting region in the world and they have encountered relatively mild resistance from rice-exporting countries other than APTERR member countries. However, if APTERR member countries agree to expand staple grain reserves beyond rice in the future, this could result in stronger opposition from other grain exporting countries.

## The necessity of incorporating food security into the CSR of transnational agribusiness

In the past few decades, transnational agribusinesses rarely involve in multilateral and regional food security mechanisms. Instead, dominant transnational food processors and commodity traders such as Archer Daniels Midland (ADM), Bunge, Cargill, and Louis Dreyfus (abbreviated as ABCD) control a fair amount of market share with extensive distribution network in the global grain trade and grain and oil seed processing industry. ABCD’s financial services divisions engage in financial transactions via derivative financial instruments in commodities futures markets, and have provided investment products to third-party investors in the last decade [60].

A food supply chain is roughly composed of producers (growers), manufacturers (processors), distributors (wholesalers), food service providers, retailers and consumers [61]. Maloni and Brown [61] developed an eight-category framework of CSR engagement in the food supply chain, namely, animal welfare, biotechnology, community, environment, fair trade, health and safety, labor and human rights, and procurement. Particularly, the eco-friendly environmental CSR category coincides with the sustainable development goal of transnational agribusinesses, which is related to the climate change mitigation measures adopted by governments towards inclusive growth. Furthermore, health and safety category particularly emphasizes the traceability of food products in the entire food supply chain to be in line with food safety and quality standards set by governments, in addition, it is required to obtain certification from a third-party organization and carry labels to ensure food safety in advance for consumers.

Transnational processors and/or retailers comply with these certification mechanisms provided by third-party organizations, such as UTZ Certified, SCS-001 and GLOBALGAP [62] and require their upstream suppliers to comply with specific standards and codes of the adopted certification to enhance food safety and quality in all aspects of production process. They do this to increase the transparency of their own CSR efforts and reputation, which increases consumers’ willingness to pay for food quality guarantee. In addition, four categories were classified by [62] from 14 independent, third-party standards and codes, including environment, labor conditions, local economy/community benefits, and food safety and quality. Each certification addresses issues slightly differently. For instance, SCS-001 requires that only organic and non-biotech food, feed and fuel be used in the production process for farmers, known as Leo-4000 in 2010 [63], but none of those SCS-001 standards and codes are involved in food security [62].

In summary, food safety and quality are the priority of food supply chain members for consumers under normal circumstances. However, the most important thing for consumers in emergencies is the quantities of adequate food available to support their basic needs. That is to say, there is no food safety without food security. From the perspective of CSR under climate change, it is worthwhile to require that transnational agribusinesses, especially food processors and commodity traders take more responsibility in food security within their business scope to keep this issue from arising in a crisis.

## Establishment of a bilateral emergency export reserve mechanism under climate change

This study proposes a framework of bilateral emergency export reserve mechanism based on multilateral and regional food security mechanisms which may incompletely adapt to the challenges of future climate change. The mechanism requires that part of a surplus of harvested grains in the grain exporting country be purchased at market prices with eligible post-harvest treatment by a transnational agribusiness, which is jointly appointed through grain exporting and importing countries, and be stored directly in local granaries of the producing country as emergency export reserves.^7^ Meanwhile, the grain importing country pays deposits for these surplus grains from a Congress-approved “food security fund” to share part of the transnational agribusiness’s storage costs. The ceiling for subscription of stored grains in the producing country is strategic grain reserves of the grain importing country (assuming 1 month). The aim of this mechanism would be to allow a way to respond either to crop failures in the grain exporting country or to food shortages occurred in the grain importing country. This provides the grain importing country with more safeguarding food security and serves as a kind of physical insurance.^8^

The principle is that a surplus of harvested grains will be purchased utilizing the cereals stocks-to-use ratio of the grain exporting country as an indicator to determine the level of crops exceeding the normal level within a certain period of time. For instance, it may be based on something as simple as determining if the cereals stocks-to-use ratio in the current year is greater than two standard deviations beyond that ratio’s moving average in the the past 5 years in the grain exporting country. However, the appropriate criteria-setting should be determined through bilateral negotiations of grain importing and exporting countries, with a goal of reducing the impact on consumers’ economic interests in the grain exporting country.

In addition, while some grain importing countries separately signed bilateral emergency export reserve agreements with the grain exporting country, the distribution of newly purchased grains for emergency export reserves for grain importing countries can be subscribed in accordance with the share of each grain importing country, that is, participating in this mechanism in the export of grain exporting country for a certain period of time. If a certain importing country renounces its right to preferentially subscribe for quotas of emergency export reserves, then the remaining grain importing countries participating in this mechanism will take over the subscription proportionally.

The timing and conditions of releasing stockpiles in emergency export reserves would be divided into two parts. First, if a grain importing country participating in this mechanism has to release its own strategic grain reserves (assuming 1 month), this signals its national grain stocks are almost exhausted. In that event, after deducting the storage loss rate^9^ of stockpiles in emergency export reserves, the transnational agribusiness would release the predetermined stockpiles of grain importing country at one time. Those edible stockpiles purchased by the grain importing country at market prices plus logistics costs are deducted from the deposits. Secondly, despite the fact that export restrictions are imposed by the grain exporting country, in compliance with the bilateral agreements signed by the signatories, the stockpiles in emergency export reserves of all grain importing countries participating in the mechanism are a one-time release.

After the edible stockpiles are sold to the grain importing country, the transnational agribusiness will repay procurement costs to the “emergency export reserve fund”. Additionally, if the transnational agribusiness makes profits from releasing those stockpiles,^10^ a previously agreed portion of profits will be used to finance crop insurance for the domestic farmers who are participating in this mechanism, thereby reducing the losses of those farmers suffering from crop failures due to natural disasters in the future.

We must also address the shelf life of stockpiles. Those which are about to expire can be made into processed foods or livestock and poultry feed and sold by the transnational agribusiness to the grain importing country, which serves as a possible source to make profits regularly for the transnational agribusiness. Both procurement costs of stockpiles and taxation on those processed foods or feed for exports could serve as supplementary sources for an “emergency export reserve fund” for the grain exporting country. If the processed foods made via this mechanism continually increase, that may place pressure on grain importing countries who must purchase those processed foods. One way to help would be for the grain exporting country to eliminate or reduce the differential export tax (DET).^11^ The WTO supports efforts to eliminate DET used to increase the competitive advantage of export products, but there is no final resolution of this issue in the draft Doha texts [14]. In addition, those stockpiles in emergency export reserves could perhaps be donated to international humanitarian organizations for humanitarian food aid with the approval of both grain importing and exporting countries.

To summarize, this mechanism has the features of reciprocity, food security enhancement and trade promotion. The expected benefits are as follows. First, transnational agribusiness purchases part of surplus grains as a source of emergency export reserves based on market prices instead of guaranteed purchase prices to reduce trade-distorting domestic support for grain production and ease the fiscal burden. Meanwhile, it also reduces the impact of lower grain prices on farmers, and mitigates the pressure of selling surplus grains abroad by imposing export subsidies by the grain exporting country in bumper harvest seasons. In addition, the grain exporting country should undertake the obligation to stabilize grain supply, benefiting from the gradual opening of the markets of grain importing countries. Transnational agribusiness can also maintain a closer relationship by serving oversees customers in their emergency needs and manifest its CSR efforts.

Secondly, the grain exporting country would establish a certain scale of emergency export reserves, with the timing of releasing those stockpiles in emergency export reserves to be determined by either the grain exporting country imposing export restrictions due to crop failures in its own country (which tightens the demand–supply gaps in world grain markets) or because of emergency needs in grain importing countries, perhaps due to natural disasters or other causes.

The advantages of emergency export reserves are illustrated below: (1) emergency export reserves can be regarded as the second line of defense for food security for the grain importing country, thus reducing the impact of any export restrictions imposed by the grain exporting country participating in the mechanism; (2) the grain exporting country would allow a one-time release of stockpiles in emergency export reserves based emergency needs on grain importing countries. This would surpass the announcement effect of improving the food security of grain importing countries [42, 43], and also contribute to reduce the risk of excessive speculation in agricultural commodities markets in poor harvest seasons; (3) transnational agribusiness may earn sales revenue by releasing stockpiles in emergency export reserves during the food crisis; and (4) the grain importing country would then have reduced incentive to diversify its countries of origin and this should also ease panic buying in emergencies.

In addition, locating storage facilities nearer to potential recipient countries will allow them to receive rapid food aid. Perhaps the location of emergency export reserves can also be nearer to rail or shipping transport facilities for faster delivery of those stockpiles, which may also shorten the response time of emergency needs from the grain importing country.

Third, the impact of climate change on the food security system of grain importing countries seems to be increasing, so the bilateral emergency export reserve mechanism would be a means of reducing the impact on food security of the grain importing country during times of turmoil in global grain trade. This may also allow international humanitarian organizations such as WFP to focus more on assisting the most vulnerable countries in emergency needs.

At the same time, the grain importing country should be willing to pay for deposits of surplus grains of grain exporting countries, even in years where they have good crops and have filled its own storage facilities. Because doing so has the merit of enhancing national food security and increasing the transparency of its grain reserves information.

Fourth, the grain importing country may purchase those stockpiles in emergency export reserves at market prices of normal years, with some added costs due to the inevitable depreciation. This will probably be at a better cost than those which must be purchased in emergencies and this has the benefit of reducing the distortion effect of G2G emergency procurement on international trade. It is worth noting that both importing and exporting countries may wish to consider signing penalty clauses for breach of contract, as well as provisions to terminate the agreement in extreme situations, rather than facing the default risks to all parties.

Finally, the bilateral emergency export reserve mechanism can be signed in the form of Bilateral Trade Agreement (BTA). This mechanism could generate a positive spillover effect for regional food security by providing increased visibility and the ability to plan on a larger scale. Due to oligopolistic structures in international grain markets and things such as transportation costs and timeliness, the number of grain exporting countries that eventually sign these clauses with a certain grain importing country may be limited. Furthermore, regional food security could be reinforced in advance through market mechanisms that allow grain exporting countries to provide their own reliable services of emergency export reserves for grain importing countries, which would give the exporters in the mechanism a competitive advantage over those who are not in the mechanism.

## Conclusion

In recent years, the world cereals stocks-to-use ratio has so far been higher than the 2007–2008 level, and regional conflicts have been limited to some areas that have not yet affected major grain production regions. These have reduced the occurrence of short-term global food crises. However, a rapid rise in trade protectionism and the latest COVID-19 pandemic have hindered the process of global trade liberalization. Continued global population growth and long-term growth in per-capita GDP coupled with limited resources, and climate change could be a substantial influence on the long-term global food supply in the future, leading to the gradual reduction of food supply as well as demand gaps. These things may well serve to increase the frequency of contemporary food crises in the future. Further, almost every country around the globe engages in economic growth and full employment as the priority for governance, so that it may be doubtful that actual carbon reduction will progress as hoped for.

It is observed that major food-exporting countries tend to inevitably impose export restrictions to protect national food security in the event of production shocks caused by climate change in the future. In addition, the impact of export restrictions will increase the risk of trade frictions between grain importers and exporters, and also the time required for grain importing countries to restore their confidence in food self-reliance. Further, faced with a food crisis, because of stagnation in the amount of in-kind assistance results in the increase in cash assistance provided by international humanitarian organizations. These couple with limited capability of regional food security systems motivate the proposal of a bilateral emergency export reserve mechanism in this paper. This would be an adaptation measure to prepare for the possibility of climate change. Transnational agribusiness would be appointed to manage a certain amount of stockpiles for emergency export reserves in the grain exporting country to strengthen grain importing country’s food security while providing more consistent markets for grain exporters. The timing of replenishing and releasing those stockpiles in emergency export reserves would be largely determined by the transnational agribusiness based on the principle of establishing a food security mechanism to meet emergency needs that may arise. This should also serve to simplify operating procedures and to increase its transparency, and more importantly to reduce the impact of trade shocks on grain importing countries participating in the mechanism.

The mutual trust and cooperation of grain importing and exporting countries participating is crucial in developing a sustainable mechanism. This mechanism can be incorporated into the bilateral trade agreement (BTA) signed by the signatories, and the activation of this mechanism requires fiscal support and congressional approval of the signatories. The maintenance of its operation depends on the management capabilities of the appointed transnational agribusiness. Therefore, responsible agencies of the signatories need to conduct regular reviews with the appointed transnational agribusiness, especially after each food crisis. Accumulating operational experience and improving deficiencies in management will help narrowing the gap between performance and goals, and make the mechanism more sustainable in responding to emergencies. Further, if this mechanism operates normally in major grain importing and exporting countries, it may serve to demonstrate that there may be an incentive for other grain importing and exporting countries to sign their respective bilateral emergency export reserve agreements under the same framework, thereby realizing bilateral cooperation for food security in practice and indirectly promoting regional food security in advance. Meanwhile, it is also a possible source of supplementary reserves to reduce the operational burden of multilateral food aid or regional food security systems.

The completeness of laws and the support of theoretical models and empirical evidence enhance the resilience of the mechanism as a prerequisite for its economic operation, therefore, future research directions are suggested as follows: (1) the clear determination of “property rights” to the emergency export reserves needs to be made to ensure that the grain exporting country will fulfill its obligations to release those stockpiles in emergency export reserves; (2) a quantitative model must be established to institutionalize and regularly adjust the optimal level of emergency export reserves to improve management efficiency; (3) a determination of the proper insurance coverage for both grain importing and exporting countries must be made to allow for emergency export reserve reinsurance; (4) to reduce storage costs and improve the emergency response capacity, the corresponding economic batch quantity in emergency export reserves can be further explored; and (5) allowing farmers to voluntarily store part of their harvested grains in emergency export reserves as well as the “grain bank”^12^ as part of this food security scheme so that good years are not wasted while balancing the income and risk of those farmers who participate in this mechanism in accordance with the national economic environment and market structure of grain exporting country.

## Data Availability

The original datasets are available in the U.S. Department of Agriculture (USDA) repository [4, 34].

## Footnotes

1 International food aid channels including multilateral, bilateral, and non-governmental organizations (NGOs) have been in operation for many years. Multilateral food aid played an essential role in the global food aid system, especially the establishment of the World Food Programme (WFP) in 1961 that accounted for approximately 60% of deliveries of the total food aid worldwide during the period 2008–2012 [16]. The United States is the dominant contributor, by far, to WFP during the last few decades. Since 2000, the amount of food aid through WFP continued to decline. This reflected the United State has reduced in-kind food aid under the concessional terms to avoid criticism from counterparties for violating fair trade practices. Meanwhile, the increased short-term emergency food aid through local and regional procurement (LRP), mainly due to improved timeliness of aid, has reduced transportation costs, met local food needs, and reduced trade distortions [16, 17].

2 Some forms of export restrictive measures listed by [14, 29] include (1) export tax (specific, ad valorem, mixed); (2) variable export tax; (3) differential export tax (DET); (4) minimum export price (MEP); (5) export quotas; (6) government to government (G2G) sales; (7) export ban or prohibition; (8) export licenses; and (9) state trading enterprise (STE).

3 During the period 2014–2019, the shares of world exports in production for rice, maize and wheat were 9.1, 13.5, and 23.5%, respectively [4, 34], so the international rice market is thinner than both maize and wheat markets.

4 Japan purchased rice from the United States as domestic rice reserves in accordance with WTO procurement practices that could only be consumed domestically, and then the United States agreed Japan to re-export its surplus WTO rice reserves to the Philippines by a means of donation or selling at cost prices [27, 43].

5 Indonesia enforced the New Food Law no. 18/2012 to increase food production and food self-sufficiency, especially the food staples as the priority to enhance national food security [46]. The Philippines proposed food staples sufficiency program 2011–2016 through production interventions to increase rice and other staples’ output [47]. China’s “No.1 central document” has been released for many years to progress farmers’ livelihoods, especially the 2014 document that highlighted national food security, ensuring the food self-sufficiency of wheat and rice through improving agricultural production capacity, and relying on food self-reliance of soybeans to meet domestic demand due to the increase in labor costs and meat consumption. Nevertheless, China’s rice imports increased substantially during the period 2012-2013, and China has been the world's top rice importer in subsequent years [3, 48].

6 http://www.apterr.org (accessed and reorganized on 31 August 2020). “*Tier 1 is formalized as a forward contract to release the earmarked emergency rice reserves for anticipated emergencies, and the payment is based on prevailing international market price. Tier 2 allows for the release of earmarked emergency rice reserves for unanticipated emergencies that cannot be addressed by Tier 1. The payment can be in cash or through long-term loan or grant. Tier 3 supports for the release of stockpiled emergency rice reserves under the contribution for severe emergencies and humanitarian aid based on the request of member countries or through automatic trigger*”[57].

7 This study suggests the establishment of a Congress-approved “emergency export reserve fund” in the grain exporting country to provide interest-free loans to the appointed transnational agribusiness to purchase surplus grains in good harvest seasons.

8 This mechanism illustrates the importance of physical stocks due to the fact that historically low world cereals stocks-to-use ratio may have been the root cause of food crises, especially during the period 2007–2008. In addition, the substantial increase in speculative positions in commodities futures markets also were an important factor in the unexpected surge in international food prices during this period [64], thus increasing the international procurement costs, financial hedging costs and operational difficulties of grain importing countries.

9 This study emphasizes that transnational agribusiness fully absorbs grain storage losses and increase the motivation to invest more in grain storage technology, thereby improving the quality of stored grains, extending the storage period, and reducing storage costs. Additionally, when global grain prices maintain a long-term upward trend and the ratio of grain prices to storage costs reaches a certain level, it would increase the incentive of grain importing countries to participate in this mechanism.

10 Although the cereals stocks-to-use ratio is an important factor in grain prices, other factors influencing grain prices include production growth rate, oil prices, US dollar exchange rates, interest rates, biofuel demand, and non-traditional hedge fund operations [10, 53, 65]. Therefore, it could only be expected that purchasing (selling) grains when the cereals stocks-to-use ratio is at a relatively high (low) level would increase the profitable opportunities of transnational agribusiness.

11 The grain exporting countries impose the differential export tax, levy a lower rate on processed products than primary products, and use lower domestic raw material prices to improve the market competitiveness of their food processing industry [14]. This measure has a subsidy effect on the export of processed products in response to tariff escalation imposed by grain importing countries.

12 Those harvested grains are stored in emergency export reserves by farmers participating in this mechanism until those batches of stockpiles are sold and payments can be received from the grain importing country via specific accounts, but those farmers have to bear their own profits and losses due to the price risk and storage costs.
